# A Mild Version of Danon Disease Caused by a Newly Recognized Mutation in the Lysosome-associated Membrane Protein-2 Gene

**DOI:** 10.7759/cureus.2155

**Published:** 2018-02-04

**Authors:** Htoo Kyaw, Fatima Shaik, Aung Naing Lin, Meir Shinnar

**Affiliations:** 1 Cardiology Department, Cardiology Fellow, Brooklyn Hospital Center; 2 Department of Internal Medicine, Mount Sinai Beth Israel Medical Center; 3 Internal Medicine, The Brooklyn Hospital Center; 4 Department of Internal Medicine, Mount Sinai Beth Israel Hospital Center

**Keywords:** danon disease, hypertrophic cardiomyopathy, lamp2 gene

## Abstract

We present the case of a patient with dilated cardiomyopathy caused by a novel mutation in the lysosome-associated membrane protein-2 (LAMP-2) gene. Patients with pathogenic mutations of this gene typically suffer from Danon disease – a condition that leads to cognitive decline, severe skeletal myopathy, and severe hypertrophic cardiomyopathy. Our patient’s presentation and clinical course, however, is different and much less severe than other patients with this disease. He did not suffer from neurologic and musculoskeletal complications. He is also possibly the longest-known survivor of this disease without a heart transplant. This disease is unfamiliar to many physicians, and our case highlights the importance of an awareness of this disorder, particularly because of its implications for both the patient and his family.

## Introduction

Danon disease (DD) was first described by Danon et al. in 1981 [1]. It is a rare X-linked dominant disorder caused by a mutation in the lysosomal-associated membrane protein-2 (LAMP-2) gene, located on chromosome Xq24 [2-3]. The LAMP-2 gene is used to synthesize a protein involved in the glycogen degradation pathway, and its defect results in glycogen accumulation mainly in the brain, muscle, and cardiac cells [3]. Therefore, DD presents with a triad of severe cardiomyopathy, skeletal myopathy, and mental retardation, of which cardiomyopathy dominates the clinical picture [3-4]. Here, we present the case of a patient with an atypical and milder version of Danon disease, i.e., cardiac manifestations in the absence of neurological and skeletal muscle pathology, caused by a previously unreported LAMP-2 gene mutation.

## Case presentation

A 46-year-old man was referred to our cardiology office in 2014. He stated that he had a history of heart problems and denied any other medical problems. He also denied any familial history of mental retardation, disorders of musculoskeletal development, cardiac disease, or sudden death. 

On a review of the records obtained from his former physician’s office, we noted that he had experienced a pre-syncopal episode in 2005. At that time, an electrocardiogram (EKG) had shown a high degree atrioventricular block, and a transthoracic echocardiogram (TTE) revealed an ejection fraction (EF) of 65%, with an intraventricular septum thickness of 3 cm. Doppler interrogation of the left ventricle outflow tract demonstrated a peak velocity of 1.4 m/sec (normal < 1.7 m/sec). He had been diagnosed with hypertrophic cardiomyopathy (HCM) while a cardiac catheterization revealed nonobstructive coronary arteries. Subsequently, he had undergone the placement of an implantable cardiac defibrillator (ICD) in 2006, as there was a significant risk of sudden cardiac death in a setting of HCM and a high degree atrioventricular block. 

He continued to follow up with a cardiologist and serial follow-up TTEs were performed. One notable feature was septal thickness, reduced to 1.6-1.9 cm in 2010 and the EF reduced to 69% in 2010 and 45%-50% in 2011. Thus, disease progression in this patient involved the thinning of the walls and transition to a dilated cardiomyopathy.

The patient was lost to follow-up for a few years after 2011. In 2014, he finally presented to our office. After a review of all the records, we considered the possibility of a genetic disease. Genetic testing for hypertrophic cardiomyopathy involved sequencing nine sarcomeric genes and three metabolic genes. The sarcomeric genes tested involved the synthesis of the following proteins – beta-myosin heavy chain, myosin-binding protein C, troponin T, troponin I, troponin C, alpha-tropomyosin, regulatory myosin light chain 2, essential myosin light chain 3, and cardiac actin. The metabolic genes tested were Anderson-Fabry disease (GLA), Danon disease (LAMP-2), and familial Wolff-Parkinson-White syndrome (PRKAG2). 

Genetic testing revealed a substitution of thymine to cytosine at position 52 (NM_002294.2, NM_013995.2(LAMP2):c.52T>C) in exon 1 of the LAMP-2 gene, resulting in a Cys18Arg mutation. A mosaic pattern was not seen. Our search led to the discovery of no other identifiable etiology of this patient’s cardiac pathology besides this genetic mutation. We thus believe that this genetic variation is in fact pathogenic.

Subsequently, the patient was informed that his cardiac condition had been traced to a genetic mutation. Since DD also involves skeletal myopathy and neurocognitive derangements, the patient was referred to a neurologist for a formal assessment of these conditions. His creatinine kinase was 404 (normal range: 30-200 U/L), but no neuromuscular abnormalities were detected on electromyographic testing. Furthermore, no cognitive defect was detected. The patient was also advised to have his family members genetically and phenotypically tested. His two children had normal results. The patient is not in touch with his parents and siblings, and, hence, they were not tested. Hence, it is unclear whether the patient has a de novo mutation or an inherited disorder. However, given the absence of any reports of a familial disease similar to that of the patient, it is quite likely that this is a de novo mutation.

**Figure 1:**
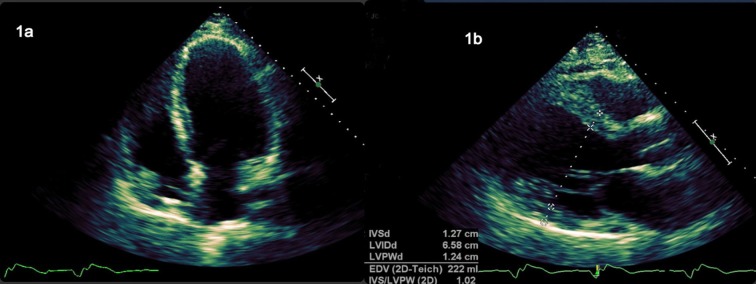
Transthoracic echocardiogram (left parasternal and apical four-chamber views) showing dilated cardiomyopathy with an interventricular thickness of 1.27 cm and an ejection fraction of 25%

In 2016, he developed exertional dyspnea during daily activities. The EKG revealed a 100% paced rhythm and the TTE showed a significantly dilated left ventricle (LVEDD of 6.6 cm, septal thickness of 1.3 cm) with an EF of 25% (Figure 1). His symptoms fell in the New York Heart Association Class 2 of congestive heart failure, guideline-directed medical therapy, including carvedilol, lisinopril, and spironolactone, were initiated. Cardiac resynchronization therapy was recommended, but the patient declined it. His symptoms have gradually improved with medical treatment and, later, lisinopril was switched to sacubitril/valsartan. Patients with Danon disease are generally referred for evaluation for heart transplantation soon after diagnosis. However, since our patient’s disease progression is much less severe and he has responded to medical therapy, we have opted to hold off on the transplantation process.

## Discussion

Our patient’s disease presentation, as well as the disease course, represents a very mild version of DD. First, he presented with the disease much later than usual. Generally, men with DD present with the disease in their late teens and women present in their late 20s. However, our patient became significantly symptomatic of the disease in his 30s.

Second, our patient did not suffer from all the complications of the clinical triad of this disease, as described earlier. He did not have any neurological manifestations of this disease. He had a mild elevation of creatinine kinase, yet, this did not translate to any form of myopathy. However, he suffered from many of the typical severe cardiac manifestations of DD. He had severe hypertrophic cardiomyopathy followed by severe dilated cardiomyopathy. This is typical of DD in that most men manifest with hypertrophic cardiomyopathy early in the disease and some of these patients do progress on to have dilated cardiomyopathy [5]. Furthermore, the patient suffered from a complete heart block, an arrhythmia seen in DD [5].

Last, DD is a rapidly progressing and fatal disease. Death before reaching the age of 25 in men is inevitable without heart transplantation [6]. However, our patient has responded well to medical therapy, and more than 10 years after diagnosis, the patient, fortunately, continues to be independent in all his activities. In fact, he may be the longest-known survivor of this disease without a cardiac transplant.

The milder version of the disease in this patient can perhaps be explained by the nature of the genetic mutation that was discovered. The patient was found to have the Cys18Arg mutation in the LAMP-2 gene. This mutation has been classified as a variant of unknown significance. It has not been described in the literature previously and is not mentioned in the genome aggregation database. Most of the previously described mutations in the LAMP-2 gene cause DD and truncal mutations that lead to a loss of the distal aspect of the protein. The mutation discovered in our patient occurs in the very proximal aspect of the gene, and its effect on the overall protein function and related clinical outcome do not seem to be as severe as the effects of previously described mutations in the LAMP-2 gene. However, the future implication of this particular mutation remains uncertain and still needs to be elucidated.

## Conclusions

This case reminds cardiologists to consider the genetic causes of cardiomyopathy whenever they encounter hypertrophic cardiomyopathy, even if the presentation does not completely fit when compared to that of the known genetic cardiac myopathies. This is particularly important for young patients, as the disease prognosis would be different and potentially quite severe.
